# Systemic Inflammation Persists the First Year after Mild Traumatic Brain Injury: Results from the Prospective Trondheim Mild Traumatic Brain Injury Study

**DOI:** 10.1089/neu.2019.6963

**Published:** 2020-09-18

**Authors:** Viktoriia Chaban, Gerard J.B. Clarke, Toril Skandsen, Rakibul Islam, Cathrine E. Einarsen, Anne Vik, Jan K. Damås, Tom E. Mollnes, Asta K. Håberg, Soeren E. Pischke

**Affiliations:** ^1^ Department of Immunology, Oslo University Hospital and University of Oslo, Oslo, Norway.; ^2^ Department of Neuromedicine and Movement Sciences, Norwegian University of Science and Technology (NTNU), Trondheim, Norway.; ^3^ Department of Radiology and Nuclear Medicine, St. Olavs Hospital, Trondheim University Hospital, Trondheim, Norway.; ^4^ Department of Physical Medicine and Rehabilitation, St. Olavs Hospital, Trondheim University Hospital, Trondheim, Norway.; ^5^ Department of Neurosurgery, St. Olavs Hospital, Trondheim University Hospital, Trondheim, Norway.; ^6^ Center of Molecular Inflammation Research, Department of Clinical and Molecular Research, Norwegian University of Science and Technology (NTNU), Trondheim, Norway.; ^7^ Department of Infectious Diseases, St. Olavs Hospital, Trondheim University Hospital, Trondheim, Norway.; ^8^ Research Laboratory, Nordland Hospital Bodø, and K.G. Jebsen TREC, University of Tromsø, Tromsø, Norway.; ^9^ Clinic for Emergencies and Critical Care, Oslo University Hospital and University of Oslo, Oslo, Norway.

**Keywords:** concussion, cytokines, growth factors, immune system, pathophysiology

## Abstract

Innate immune activation has been attributed a key role in traumatic brain injury (TBI) and successive morbidity. In mild TBI (mTBI), however, the extent and persistence of innate immune activation are unknown. We determined plasma cytokine level changes over 12 months after an mTBI in hospitalized and non-hospitalized patients compared with community controls; and examined their associations to injury-related and demographic variables at admission. Prospectively, 207 patients presenting to the emergency department (ED) or general practitioner with clinically confirmed mTBI and 82 matched community controls were included. Plasma samples were obtained at admission, after 2 weeks, 3 months, and 12 months. Cytokine levels were analysed with a 27-plex beads-based immunoassay. Brain magnetic resonance imaging (MRI) was performed on all participants. Twelve cytokines were reliably detected. Plasma levels of interferon gamma (IFN-γ), interleukin 8 (IL-8), eotaxin, macrophage inflammatory protein-1-beta (MIP-1β), monocyte chemoattractant protein 1 (MCP-1), IL-17A, IL-9, tumor necrosis factor (TNF), and basic fibroblast growth factor (FGF-basic) were significantly increased at all time-points in patients compared with controls, whereas IFN-γ-inducing protein 10 (IP-10), platelet-derived growth factor (PDGF), and IL-1ra were not. IL-17A and FGF-basic showed significant increases in patients from admission to follow-up at 3 months, and remained increased at 12 months compared with admission. Interestingly, MRI findings were negatively associated with four cytokines: eotaxin, MIP-1β, IL-9, and IP-10, whereas age was positively associated with nine cytokines: IL-8, eotaxin, MIP-1β, MCP-1, IL-17A, IL-9, TNF, FGF-basic, and IL-1ra. TNF was also increased in those with presence of other injuries. In conclusion, mTBI activated the innate immune system consistently and this is the first study to show that several inflammatory cytokines remain increased for up to 1 year post-injury.

## Introduction

The World Health Organization (WHO) has identified traumatic brain injury (TBI) as a key societal challenge as it emerges as the number one cause of death and disability from 2020, with 2.5 million cases estimated to occur each year in the European Union.^1^ More than 85% of TBIs are classified as mild.^2^ Although the majority recover well after mild TBI (mTBI), 10–20% experience long-lasting symptoms such as headache, emotional distress, and problems with concentration and memory.^3,4^

Given that even mild tissue injury can damage cells and release or expose damage associated molecular patterns (DAMPS), we can hypothesize that inflammation in the brain, which is detectable in systemic blood, could be an important pathophysiological mechanism following mTBI.^5^

The interplay between central nervous system (CNS)-derived inflammation and systemic inflammation is intricate. Recent evidence suggests that cytokines orchestrate a complex interplay between peripheral leukocytes and chronically activated microglia, especially if the blood–brain barrier is compromised after mechanical damage, as seen in TBI.^6^ Although activation of the innate immune system is crucial for recovery after TBI, as it promotes beneficial clearance of injured cells/cell debris, prolonged neuroinflammation has been shown to be detrimental, leading to progressive CNS degeneration.^5,7^ In experimental models, it was shown that repeated mTBI caused systemic and neuroinflammation associated with chronic behavioral deficits.^8^ In sports-related mTBI an immediate activation of the innate immune system (interleukin 6 [IL-6], IL-12) has been observed with return to baseline after a week.^9^ The long-term course of systemic inflammation and type of cytokines present in blood in patients with mTBI has been evaluated sparsely, with one study including 52 patients showing an increase of IL-1β, IL-6, and monocyte chemoattractant protein 1 (MCP-1) over a 3-month period with association of MCP-1 to post-concussion syndrome.^10^

Many patients with mild TBI are not admitted to hospitals and are thus possibly underrepresented in clinical studies.^11^ Therefore, the Trondheim Mild TBI study was designed to include patients who were seen in the primary care setting for mTBI, in addition to patients who were admitted to the hospital, with 1-year follow-up of all patients.^12^ In addition, age-matched community controls were included for the same length of the follow-up for comparison.

In the present study, we examined plasma levels of a wide range of inflammatory biomarkers in patients with mTBI from admission to 12 months after injury compared with age-, sex-, and education-matched community controls over the same time period. We then examined associations between injury-related and demographic variables and cytokine levels in the acute phase.

We hypothesized that mTBI leads to increases in systemic cytokines over a prolonged time period and that acute-phase inflammatory cytokines are associated with injury-related and demographic factors.

## Methods

### Ethics, participants, and recruitment

The Trondheim Mild TBI study is a large-scale prospective cohort study with follow-up for 12 months in patients with mTBI and matched controls, all between 16 and 59 years of age (Norwegian National Ethics Approval: REK 2013/754). The upper age limit was chosen due to the higher frequency of comorbidities in the elderly. All participants, and guardians of participants between the age of 16 and 18 years, provided informed consent. The cohort and magnetic resonance imaging (MRI) follow-up have been described in detail previously.^12,13^ All data were handled in accordance with the STROBE checklist (Supplementary Fig. S1).

Patients were included in the study between April 1, 2014 and December 5, 2015. Patients were prospectively recruited by continuous screening of computed tomography (CT) referrals and patient lists from two emergency departments (EDs): St. Olavs Hospital (Trondheim University Hospital), a regional Level 1 trauma center in Trondheim, Norway, and Trondheim Municipal Emergency Clinic, a general practitioner-run, outpatient clinic. Patients were included when having sustained a TBI categorized as mild according to the WHO criteria: 1) Glasgow Coma Scale (GCS) score 13–15 at presentation in the ED; 2) witnessed loss of consciousness (LOC) <30 min, confusion, or post-traumatic amnesia (PTA) <24 h, or traumatic lesion on neuroimaging, and did not meet any exclusion criteria. (Supplementary Table S1).^14,15^

Clinical information was obtained from patient interviews and medical records. LOC was rated as present only if observed. Duration of PTA was recorded as the time after injury for which the patient had no continuous memory (< 1 h, or 1–24 h). GCS score was assessed in the ED or inferred from the record. Presence of injuries to parts of the body other than the head was recorded, and dichotomized into “yes” or “no,” based on self-report and records. Such injuries were cranial fractures; fractures of extremities, clavicles, and ribs; wounds; and sprains, dislocations, and other soft-tissue injuries. Bruises and wounds that did not need suturing were not included. Major trauma was an exclusion criterion in the study.

Community controls, matched on age, sex, and years of education were recruited among staff, friends, and families of staff and patients. The same exclusion criteria as for the TBI patients were used (Supplementary Table S1).

### Magnetic resonance imaging protocol

MRI scans were acquired using a 3.0 Tesla Siemens Skyra MRI scanner, software version E11C, with a 32-channel head coil. The same MRI protocol was used for all participants; three-dimensional (3D) volumes were obtained with T1-weighted (magnetization prepared rapid acquisition gradient echo [MPRAGE]), T2-weighted, fluid-attenuated inversion recovery (FLAIR), and susceptibility-weighted imaging (SWI). An axial (2D) diffusion-weighted scan and a diffusion tensor/kurtosis scan were also acquired. The clinical scans were read by an experienced neuroradiologist according to predefined criteria as described in a previous publication.^13^

### Blood samples

Blood was acquired from patients with mTBI in the admission phase, defined as within 72 h post-injury; 2 weeks (± 3 days); 3 months (± 2 weeks); and 12 months (± 1 month) after the injury. For the community controls, blood was collected at inclusion corresponding to the admission phase for the patients, and after 3 and 12 months. Plasma samples used in the current study were obtained from whole blood collected in 5-mL tubes containing ethylenediaminetetraacetic acid (EDTA), directly placed on ice, and centrifuged within 30 min at 2000 x *g* for 10 min at 4°C. Aliquoted plasma samples were immediately stored at −80°C.

### Cytokine analysis

The EDTA plasma samples were analyzed using a commercial fluorescence magnetic bead-based immunoassay, with high-sensitivity detection range and precision (Bio-Plex Human Cytokine 27-Plex, Bio-Rad Laboratories, Inc., Hercules, CA, USA). The following cytokines were analyzed: IL-1β, IL-1 receptor antagonist (IL-1ra), IL-2, IL-4, IL-5, IL-6, IL-7, IL-8 (C-X-C motif chemokine ligand 8; CXCL8), IL-9, IL-10, IL-12, IL-13, IL-15, IL-17, eotaxin-1 (C-C motif chemokine ligand 11; CCL11), basic fibroblast growth factor (FGF-basic), granulocyte colony stimulating factor (GCSF), granulocyte-macrophage colony stimulating factor (GM-CSF), interferon gamma (IFN-γ), IFN-γ-inducing protein 10 (IP-10; CXCL10), monocyte chemoattractant protein 1 (MCP-1; CCL2), macrophage inflammatory protein-1-alpha (MIP-1α; CCL3), macrophage inflammatory protein-1-beta (MIP-1β; CCL4), platelet-derived growth factor-BB (PDGF-BB), RANTES (CCL5), tumor necrosis factor (TNF), and vascular endothelial growth factor (VEGF).

The analyses were performed according to the manufacturer's instructions. Briefly, plasma samples were diluted 1:4 in Sample Diluent (Bio-Rad Laboratories, Inc.). A lower detection limit for the cytokines in the low picogram/milliliter range (<20 pg/mL for all cytokines) was determined automatically by the software based on the standard curve for each cytokine. Based on many years of experience with the multi-plex assay and a low inter-assay coefficient of variation (<11 for all cytokines), the samples were run in single. All samples from this study were analyzed using the same batch of the Bio-Plex assay and on each plate randomly chosen sample sets from patients and healthy controls were analyzed.

Under physiological conditions most of the cytokines in plasma are either not detected or detected in very low amounts.^16^ Thus, only cytokines that were present in methodologically and clinically meaningful amounts, according to our previous experience,^16^ in more than 75% of all samples during the observation period, were selected for further study (*n* = 12, see Results section). The remaining 15 were regarded as negative and therefore not included in further analyses.

### Statistical analysis

Frequencies and percentages of demographic and clinical variables for the total number of participants with data available at a minimum of one time-point were calculated.

Descriptive statistics (mean, standard deviation, median, interquartile range, and range) of non-log transformed cytokine values were calculated.

Cytokine data are presented as box plots. Mixed model analyses were performed to compare the time course of cytokine levels from the admission phase to 12 months after injury for the mTBI group versus the community control group. As there are no available control data at the 2-week time-point, this time-point was removed from the mixed model analyses, although the 2-week patient data are retained in the box plots. Certain cytokine concentrations were log-transformed due to large ranges and non-normal distribution of the data (marked on plot legends and table legends). The mixed model analyses were conducted with time, group, and time-by-group interaction as fixed effects and a subject-specific random intercept to account for within-subject correlations. Mixed model analyses were performed for all cytokines with and without controlling for heterogeneous variances. All model fits were shown to be improved with heterogeneous variances controlled for (Supplementary Table S2), thus all data presented are derived from these models. Post hoc contrast analyses between patient and control groups were performed for all cytokines that showed a main effect of group. For those showing a time-by-group interaction effect, further post hoc contrast analyses were used to assess within-group changes for the mTBI group between successive time-points.

Best-subset regression analyses were performed to determine the combination of demographic (sex and age) and clinical (GCS score, PTA duration, LOC, traumatic MRI findings) variables that best predict admission-phase cytokine levels. The best model was determined based on the lowest Akaike Information Criterion (AIC). The cytokine values were first standardized at each time-point, and then the best-subset regression analyses were performed using the standardized biomarkers as outcome variables. Regression coefficients based on non-standardized biomarker values were also reported. In the patients with mTBI, we also calculated the empirical group means and standard deviations of cytokine levels for each categorical predictor used in the best-subset regression analysis.

All tests were two-sided with significance determined at *p* < 0.05. Post hoc contrast analyses of the mixed models were Bonferroni corrected (significance level of group differences at each time-point: 0.05/3 = 0.017; significance level of within-group changes for the mTBI group between time-points: 0.05/3 = 0.017).

All statistical tests were calculated using R version 3.2.2.^17^ Descriptive statistics were calculated using R's base package functions. Mixed model analyses were performed using the nlme package.^18^ Best-subset multiple regressions were performed using the bestglm package.^19^

## Results

### Study cohort

**FIG. 1.**
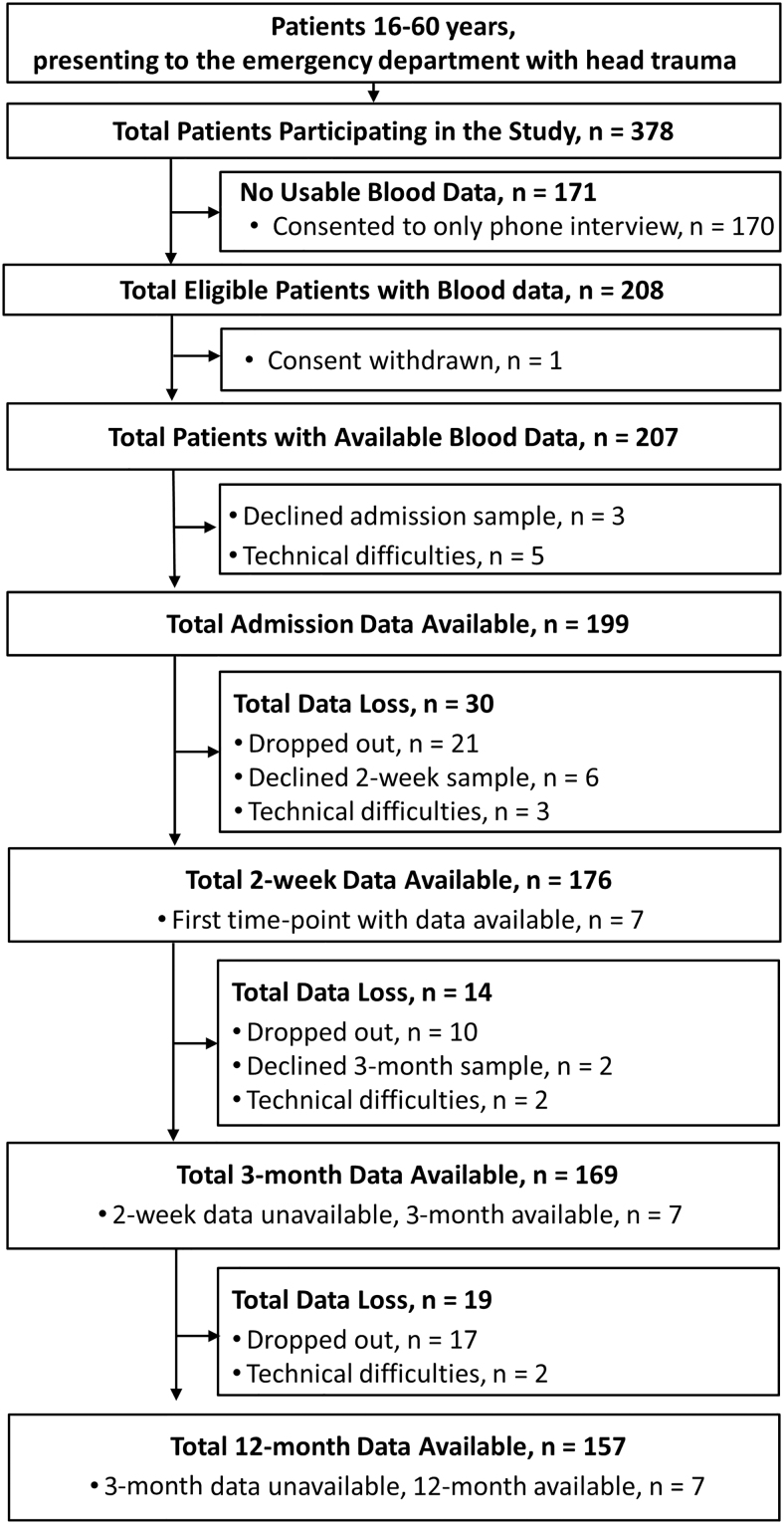
Identification, enrollment, and follow-up of patients with mild TBI (mTBI). Flow chart of inclusion of patients with mTBI into the Trondheim Mild TBI follow-up study, along with progression of blood data available at each successive time-point. CT, computed tomography; mTBI, mild traumatic brain injury.

**FIG. 2.**
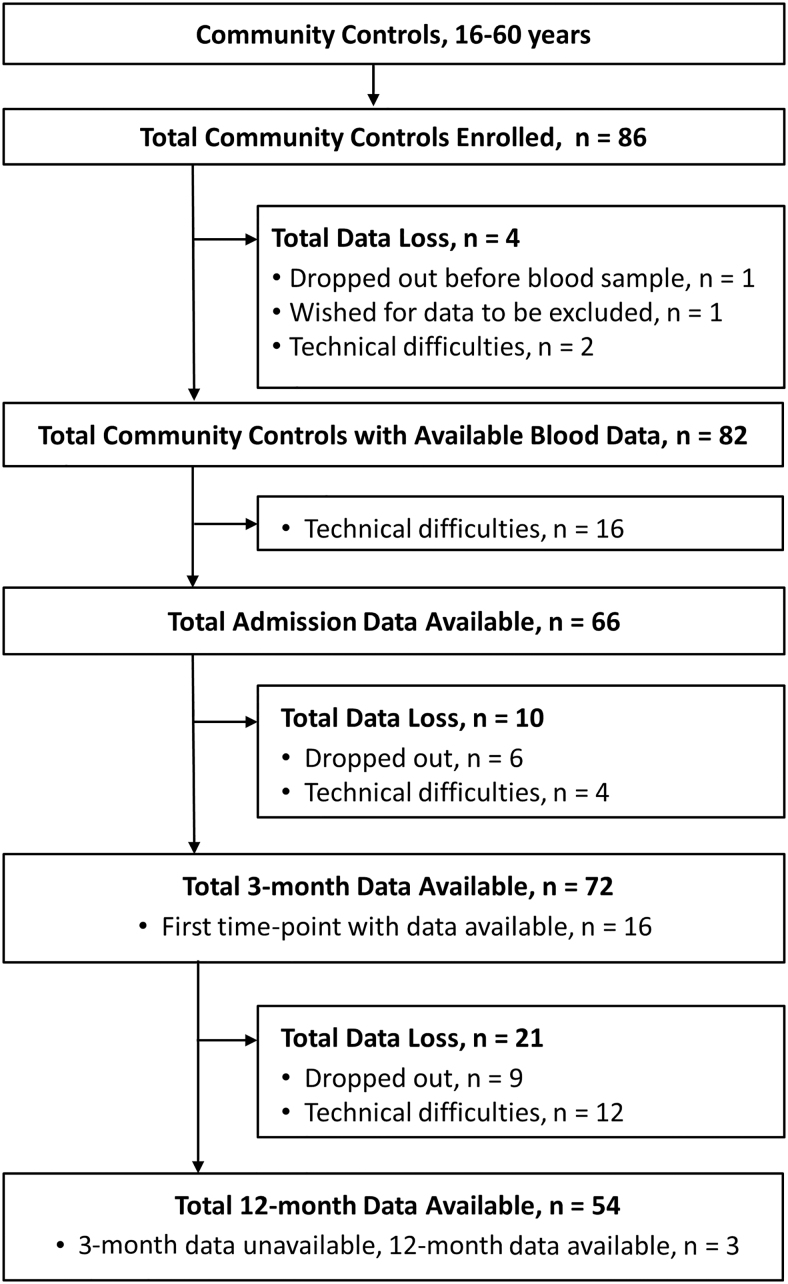
Identification, enrollment, and follow-up of community controls. Flow chart of inclusion of community controls along with progression of blood data available at each successive time-point. Blood samples were drawn from community controls at enrollment (admission) and 3-month and 12-month time-points (not 2 weeks).

In total, 379 patients with mTBI participated in the overall study. Blood samples were provided from 207 patients for at least one time-point and of these, 194 had brain MRI performed within 72 h. (Fig. 1). Of the 86 community controls enrolled, 82 provided blood samples for at least one time-point (Fig. 2).

Demographic and injury-related characteristics of the mTBI and control group showed that 63.3% of patients with mTBI were male (Table 1). The majority had very mild injuries, with GCS scores of 15 in 76%; 52.7% were not observed with a LOC, most had PTA of less than 1 h (69.1%), intracranial traumatic findings MRI at 72 h were present in few (11.1%), and the majority (63.3%) did not experience other injuries (Table 1). The most common other injuries were soft-tissue injuries.

**Table 1.** Patient Characteristics

	Patients with mild TBI Patients	Controls
	*N* = 207	*N* = 82
Gender (%)		
Males	131 (63.3)	46 (56.1)
Females	76 (36.7)	36 (43.9)
Age at inclusion		
Mean age, years (SD)	32.4 (13.2)	33.02 (12.9)
Age range, years	16 – 60	16 – 60
GCS (%)		
13	5 (2.4)	
14	33 (16.0)	
15	158 (76.0)	
Unknown	11 (5.3)	
LOC (%)		
Unobserved LOC	109 (52.7%)	
Observed LOC	98 (47.3%)	
PTA (%)		
PTA <1 h	143 (69.0)	
PTA 1-24 h	64 (31.0)	
Traumatic intracranial finding on MRI at 72 h (%)		
TAI only	6 (2.9)	
Contusion only	3 (1.4)	
Intracranial hematoma only	3 (1.4)	
TAI and contusion	5 (2.4)	
Contusion and hematoma	6 (2.9)	
No findings	184 (88.9)	
Other injuries (%)		
None	131 (63.3)	
Fractures	35 (16.9)	
Soft-tissue injuries	41 (19.8)	

Total number with percentages in parenthesis presented.

GCS, Glasgow Coma Score; LOC, loss of consciousness; MRI, magnetic resonance imaging; PTA, post-traumatic amnesia; SD, standard deviation, TAI, traumatic axonal injury.

### Time course of plasma cytokine levels in patients with mTBI from admission to 12 months after injury compared with controls

**FIG. 3.**
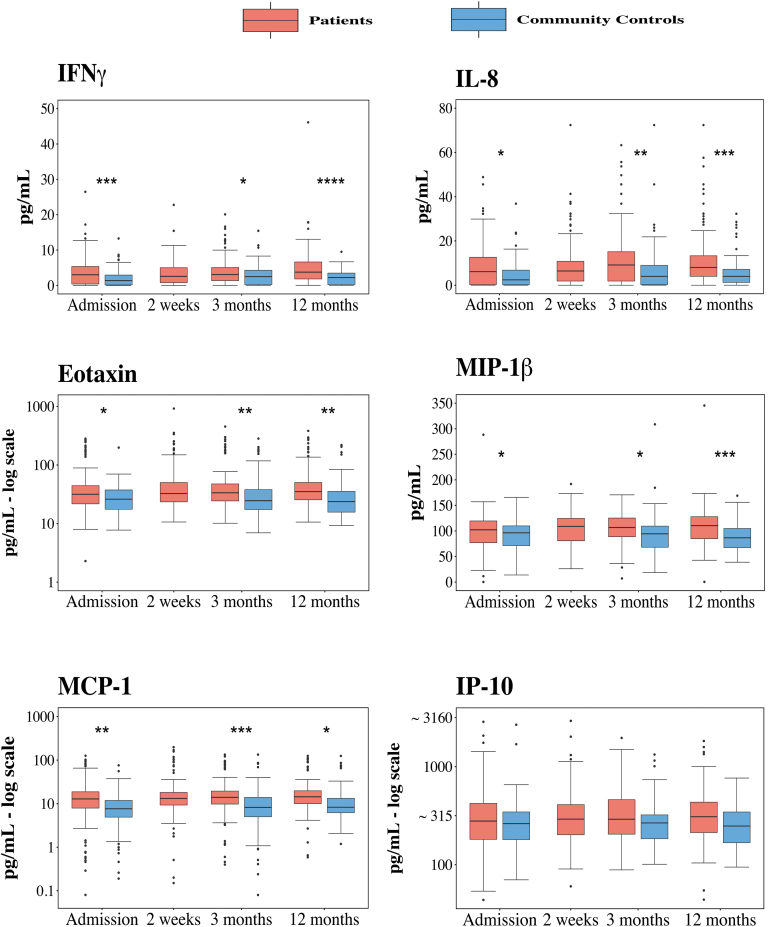
Levels of IFNγ and five chemokines for patients with mTBI and controls over time. IFNγ, IL-8, eotaxin, MIP-1β, and MCP-1 were significantly higher in patients than in community controls at all time-points. IP-10 was not significantly different between groups at any time-point. Data are presented as box plots with median as line, borders, 25th and 75th percentile, and whiskers (value of the 25th and 75th percentile +1.5 interquartile range). Points above and below the whiskers represent outliers. Asterisks (*) indicate significant group difference between patients and controls at a particular time-point in the linear effect model. The *p*-value level is represented as follows:* <0 .05, ** <0.01, *** <0.001, **** <0.0001. IFN-γ, interferon gamma; IL-8, interleukin 8; IP-10, IFN-γ-inducing protein 10; MCP-1, monocyte chemoattractant protein 1; MIP-1β, macrophage inflammatory protein-1-beta; mTBI, mild traumatic brain injury. Color image is available online.

**FIG. 4.**
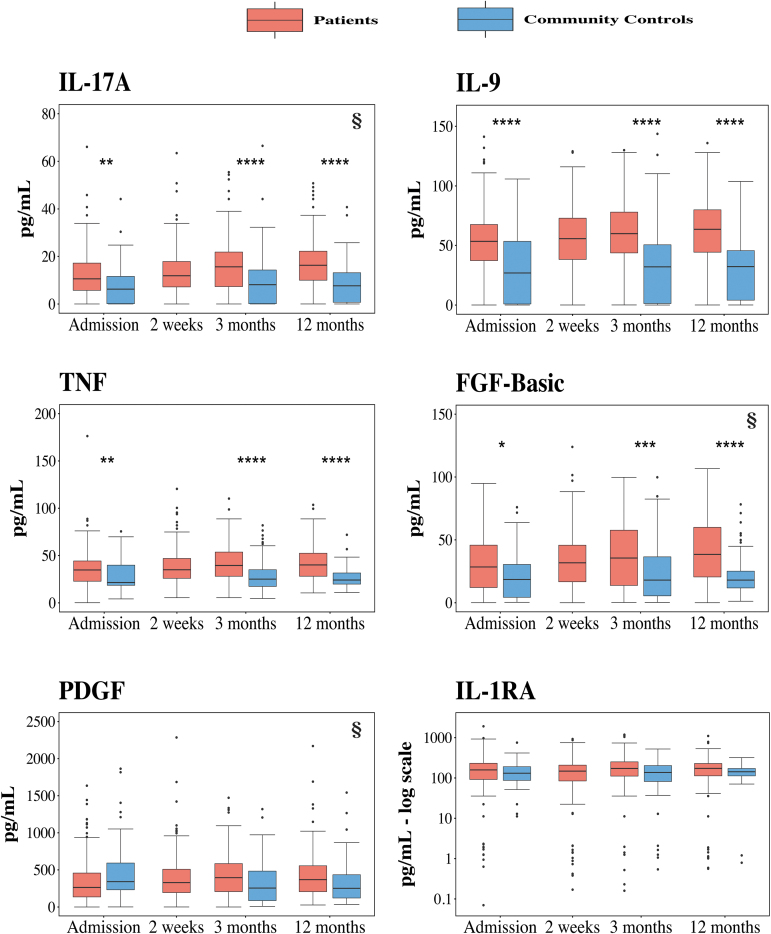
Levels of interleukins, TNF, growth factors, and IL-1ra for patients with mTBI and controls over time. IL-17A, IL-9, TNF, and growth factor FGF-basic were significantly higher in patients than in community controls at all three time-points. No differences were shown for growth factor PDGF and regulator IL-1ra. For IL-17A, FGF-basic, and PDGF, the group differences between patients with mTBI and community controls increased throughout the 12-month observation period (§). Box plots, outliers, and group differences are presented and calculated in the same manner as in Figure 3. §, significant time x group interaction in the linear effect model; FGF-basic, basic fibroblast growth factor; IL, interleukin; IL-1ra, IL-1 receptor antagonist; mTBI, mild traumatic brain injury; PDGF, platelet-derived growth factor; TNF, tumor necrosis factor. Color image is available online.

Cytokines were grouped according to biological function or class; pro-inflammatory IFNγ and chemokines (Fig. 3), pro-inflammatory interleukins (Fig. 4A–C), growth factors (Fig. 4D,E), and anti-inflammatory regulator IL-1ra (Fig. 4F). Nine of the twelve cytokines assessed showed significant differences between the mTBI patient and control groups, whereas IP-10, PDGF, and IL-1ra did not show an effect of group (Table 2). For the cytokines showing a significant effect of group, contrast analyses to assess group differences at each time-point revealed significant differences between patients with mTBI and controls at each time-point assessed (Fig. 3 and 4, Table 3). A time-by-group interaction was evidenced for FGF-basic and IL-17A, indicating that the time course between patients and controls differed for those two cytokines. Five of the cytokines (MIP-1β, MCP-1, TNF, IL-9, and IL-8) showed significant differences of both group and time, but no time-by-group interaction. PDGF showed a significant interaction of time-by-group, but no main effect of group or time. Two cytokines (IP-10 and IL-1ra) demonstrated no effects of group or time, nor a time-by-group interaction (Table 2).

**Table 2.** Summary of Main Effects of Group and Time and the Interaction of Group and Time for Each of the 12 Cytokines

Cytokines	Group F-value (*p*-value)	Time F-value (*p*-value)	Interaction (group-by-time) F-value (*p*-value)
IFN-γ	F = 22.42, ***p* < 0.0001**	F = 1.55, *p* = 0.213	F = 2.99, *p* = 0.051
IL-8	F = 10.64, ***p* = 0.001**	F = 7.83, ***p* = 0.0005**	F = 2.00, *p* = 0.136
Eotaxin^a^	F = 8.90, ***p* = 0.003**	F = 2.66, *p* = 0.071	F = 1.57, *p* = 0.210
MIP-1β	F = 10.91, ***p* = 0.001**	F = 4.34, ***p* = 0.014**	F = 0.96, *p* = 0.382
MCP-1^a^	F = 9.44, *p* = 0.**002**	F = 3.34, *p* = 0**.037**	F = 1.87, *p* = 0.155
IP-10^a^	F = 3.75, *p* = 0.054	F = 0.82, *p* = 0.442	F = 0.05, *p* = 0.954
IL-17A	F = 20.59, ***p*< 0.0001**	F = 14.38, ***p*< 0.0001**	F = 4.06, ***p* = 0.018**
Il-9	F = 29.66, ***p*< 0.0001**	F = 10.00, ***p* = 0.0001**	F = 0.17, *p* = 0.842
TNF	F = 21.47, ***p*< 0.0001**	F = 6.30, ***p* = 0.002**	F = 1.05, *p* = 0.351
FGF-basic	F = 17.40, ***p*< 0.0001**	F = 14.67, ***p*< 0.0001**	F = 4.97, ***p* = 0.007**
PDGF	F = 2.80, *p* = 0.095	F = 1.35, *p* = 0.261	F = 4.99, ***p* = 0.007**
IL-1ra^a^	F = 0.36, *p* = 0.698	F = 0.50, *p* = 0.481	F = 2.45, *p* = 0.088

^a^ Log transformed data. Significant differences are bolded.

^a^ Linear mixed model analysis.

Group: patients with mild TBI compared with community controls when time is not taken into account; Time: time course of cytokine concentrations when group is not taken into account; Group-by-Time: interaction of Group and Time; a significant effect indicates the time courses of patients with mild TBI and community controls significantly differed.

FGF-basic, basic fibroblast growth factor; IL, interleukin; IL-1ra, IL-1 receptor antagonist; IFN-γ, interferon gamma; IP-10, IFN-γ-inducing protein 10; MCP-1, monocyte chemoattractant protein 1; MIP-1β, macrophage inflammatory protein-1-beta; PDGF, platelet-derived growth factor; TBI, traumatic brain injury; TNF, tumor necrosis factor.

**Table 3.** Group Comparisons between Patients with Mild TBI and Community Controls at Each Time-point, for Each of the Cytokine Concentrations Showing a Significant Group Effect in the Previous Analysis

	Admission estimate^a^ [95% CI] *p*-value	3 months estimate^a^ [95% CI] *p*-value	12 months estimate^a^ [95% CI] *p*-value
IFN-γ	1.4 [0.6 – 2.2]	1.0 [0.1 – 1.9]	2.3 [1.4 – 3.1]
	***p* = 0.0007**	*p* = 0.023	***p*< 0.0001**
IL-8	2.8 [0.04 – 5.5]	4.2 [1.2 – 7.1]	4.8 [2.2 – 7.4]
	*p* = 0.047	***p* = 0.006**	***p* = 0.0003**
Eotaxin^b^	**0.1** [0.02 – 0.2]	**0.1** [0.05 – 0.2]	**0.1** [0.05 – 0.2]
	***p* = 0.017**	***p* = 0.002**	***p* = 0.002**
MIP-1β	9.3 [0.5 – 18.0]	11.5 [2.6 – 20.4]	14.4 [6.4 – 22.3]
	*p* = 0.038	***p* = 0.012**	***p* = 0.0004**
MCP-1^b^	0.19 [0.06 – 0.32]	0.25 [0.010 – 0.329]	0.14 [0.03 – 0.26]
	***p**** =* **0.04**	***p**** =* **0.0008**	***p**** =* **0.013**
IL-17A	3.7 [1.1 – 6.4]	5.5 [2.8 – 8.2]	6.6 [4.1 – 9.1]
	***p* = 0.006**	***p*< 0.0001**	***p*< 0.0001**
IL-9	19.4 [11.3 – 27.4]	20.2 [12.4 – 28]	20.9 [13.2 – 28.5]
	***p*< 0.0001**	***p*< 0.0001**	***p*< 0.0001**
TNF	8.0 [3.0 – 13.0]	10.7 [6.1 – 15.4]	10.5 [6.0 – 15.1]
	***p* = 0.002**	***p*< 0.0001**	***p*< 0.0001**
FGF-basic	7.3 [1.3 – 13.3]	10.2 [4.4 – 16.0]	14.4 [8.8 – 20.1]
	***p* = 0.017**	***p* = 0.0006**	***p*< 0.0001**

^a^ Estimate refers to mean group differences as estimated by the mixed model. CI is the 95% confidence interval of the estimated group difference.

^b^ Log transformed data. Post hoc contrast analyses based on the linear mixed model. Significant differences are bolded.

FGF-basic, basic fibroblast growth factor; IL, interleukin; IFN-γ, interferon gamma; MCP-1, monocyte chemoattractant protein 1; MIP-1β, macrophage inflammatory protein-1-beta; TBI, traumatic brain injury; TNF, tumor necrosis factor.

Lastly, for the cytokines IL17-A and FGF-basic, which showed both a significant group effect and a significant time-by-group interaction, post hoc contrast analyses were performed to assess differences between time-points for patients with mild TBI (Table 4). Both increased between admission and 3 months; however, there was no significant increase between 3 and 12 months. Significant differences were observed also between admission and 12 months, indicating patient cytokine levels did not return to control levels at 12 months.

**Table 4.** Comparisons between Time-Points in Patients with Mild TBI, for Each of the Cytokine Concentrations Showing a Significant Group Effect and a Significant Group-by-Time Interaction

	Admission – 3 months Estimate^a^ [95% CI] *p*-value	3 months – 12 months Estimate^a^ [95% CI] *p*-value	Admission – 12 months Estimate^a^ [95% CI] *p*-value
IL-17A	3.7 [2.3 – 5.2]	-0.2 [-1.8 – 1.4]	3.5 [2.0 – 5.0]
	***p*< 0.0001**	*p* = 0.796	***p*< 0.0001**
FGF-basic	7.3 [4.2 – 10.4]	2.5 [-1.0 – 5.9]	9.8 [6.4 – 13.2]
	***p*< 0.0001**	*p* = 0.157	***p*< 0.0001**

^a^ Estimate refers to mean group differences as estimated by the mixed model. CI is the 95% confidence interval of the estimated group difference.

Post-hoc contrast analyses based on the linear mixed model.Significant differences are bolded.

FGF-basic, basic fibroblast growth factor; IL, interleukin; TBI, traumatic brain injury.

### Association of cytokine levels at admission with demographic and injury-related variables

**FIG. 5.**
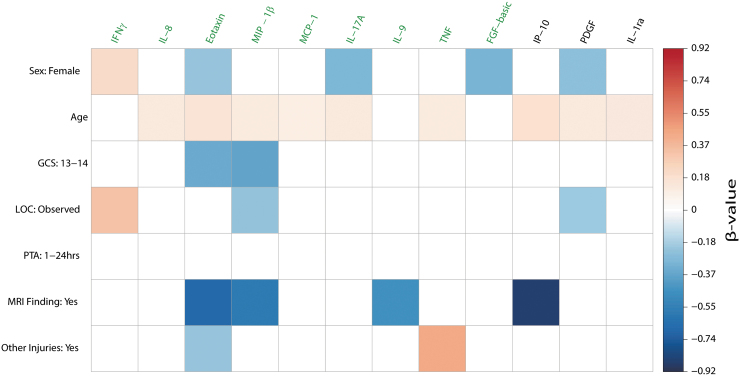
Cytokines at admission are associated with demographic and clinical variables. Cytokines were input into an all-subsets multiple regression as outcome variables, and all demographic and clinical variables were input as possible predictor variables. Separate regressions were performed for each cytokine, with the associations shown to improve model fit depicted as colored squares. Model fit was determined based on the Akaike Information Criterion. Associations presented are the beta-values of the final model. A blank space indicates that the predictor was not included in the final model. The direction and size of regression coefficient is represented according to the figure legend color scheme, whereby increasingly positive associations are graded to red, and increasingly negative associations are graded to blue. The nine cytokines colored in green correspond to those whose linear mixed models revealed a significant group difference between patients and controls, whereas the three cytokines to the right (black) did not show significant group differences. All beta-values are standardized. Non-standardized beta-values and *p*-values are presented in Supplementary Table S3. Baseline comparison for Sex was male. Baseline comparison for GCS scores of 13–14 was a GCS score of 15. Baseline comparison for Observed LOC was “Unobserved LOC.” Baseline comparison for PTA duration of between 1 and 24 h was PTA of less than 1 h. Baseline comparison for MRI findings was “No MRI Finding.” Baseline comparison for presence of injuries to other parts of the body was “No Other Injuries.” GCS, Glasgow Coma Score; LOC, loss of consciousness; MRI, magnetic resonance imaging; PTA, post-traumatic amnesia. Color image is available online.

Associations between the 12 cytokines at time of admission and demographic and clinical predictors were calculated in an all-subset multiple regression analysis with standardized beta coefficients (Fig. 5). Non-standardized beta coefficients and *p*-values are presented in Supplementary Table S3 and exact values of cytokines in Supplementary Table S4. The models of eotaxin, MIP-1β, IL-9, and IP-10 included negative associations of MRI findings, meaning those with MRI findings exhibited lower levels of inflammatory markers than those with no MRI findings.

Age was included in the models for the majority of cytokines, specifically: IL-8, eotaxin, MIP-1β, MCP-1, IL-17A, TNF, IL-1ra, IP-10, and PDGF. The associations were positive for all cytokines, indicating that inflammation markers increase as a function of increasing age.

“Other injuries” showed a strong positive effect on TNF, which indicates that TNF levels are significantly higher in those with injuries to other parts of the body. To ensure the group difference between mTBI and controls (Fig. 3) was not due to other injuries, we re-ran the time course linear mixed model including mTBI patients without other injuries versus controls. The mTBI group without other injuries had increased TNF levels compared with the controls at 3 and 12 months, and a significant group and time effect was present (Supplementary Tables S5 and S6). The increased TNF level in the mTBI group in the acute phase was thus mainly due to other injuries. But, during the first year TNF levels increased significantly in the mTBI group without other injuries. These results show that mTBI per se was associated with chronically elevated TNF levels.

A negative effect of other injuries was also included in the model for eotaxin. Re-running the time course linear mixed model with the mTBI patients without other injuries versus controls uncovered that the mTBI group without other injuries contributed most to the increased eotaxin levels in the acute phase. The mTBI patients without other injuries had significantly higher eotaxin levels than controls at all 3 time-points, and a significant group effect was present (Supplementary Tables S5 and S6). The increased eotaxin levels in mTBI can therefore be ascribed to the TBI alone.

LOC was included as a positive association in the IFNγ model, but a negative association in the MIP-1β and eotaxin models, indicating that in those with observed LOC IFNγ concentrations were higher, whereas MIP-1β and eotaxin concentrations were lower. GCS scores were included as negative associations in models of eotaxin and MIP-1β, indicating those with lower GCS score (i.e., more severe injury) exhibited lower cytokine values. Lastly, sex was included as a positive association in only the IFNγ model, and as a negative association in the eotaxin, IL-17A, FGF-basic, and PDGF models. This indicates that female patients with mTBI exhibited higher values of IFNγ and lower values of the other four cytokines mentioned above. PTA was not included in any of the cytokine models.

## Discussion

In this unique cohort of patients with mTBI, treated both in the hospital and the primary health care setting, we showed a prolonged increase in cytokines in blood, reflecting an inflammatory response from admission to 1 year after mTBI compared with matched healthy controls.

### Prolonged activation of the systemic immune system in mTBI

In contrast to previous studies investigating limited numbers of cytokines, we used an unbiased assessment of 27 cytokines.^10^ Cytokines form part of a complex inflammatory network,^20^ which has been shown to be associated with injury severity in moderate to severe TBI, in both human and experimental studies.^21,22^ Our study is, to the best of our knowledge, the first to show significant systemic cytokine elevations persisting for 1 year in mTBI across a pro-inflammatory cytokine network.^23,24^

To date, studies of cytokine expression patterns over time have focused on patients with moderate to severe TBI demonstrating slightly higher plasma concentrations in the acute phase post-injury than observed in our study.^22,24^ Whereas long-term plasma cytokine studies, to our knowledge, are lacking in patients with severe TBI, autopsy studies have revealed that intracerebral inflammation leading to microglial activation can persist for years, and we can thus only speculate that this inflammation would be observable in plasma as well, comparable to patients with mTBI in this study.^7^ In this study in mTBI, cytokine plasma levels were generally low and lower than observed during severe TBI.^24^ However, persistent, low-grade inflammation, comparable to the cytokine levels obtained in this study, has been described in one prior study of patients with mTBI and chronic diseases with inflammatory components such as Alzheimer's and multiple sclerosis, and has been attributed to long-term morbidity, such as fatigue and mortality.^10,25,26^

A hallmark in this study is the prolonged activation of cytokines over the 1-year follow-up period in patients with mTBI, compared with matched community controls. This is in contrast to studies showing acute but transient increase of plasma cytokines in mTBI. In a study of blast-exposed military personnel, cytokines IL-6 and TNF returned to baseline levels within hours,^27^ whereas in sports-related concussion cytokines IL-6 and IL-12 returned to baseline within 7 days.^9^ In children with concussion, TNF, IL-6, IL-1β, IL-10, and several neuronal damage plasma markers returned to baseline within days, whereas IL-8 remained increased for 3 months.^28^ Likewise, a recent study in 52 patients with mTBI showed prolonged increase of IL-1β, IL-6, and MCP-1, combined with a general increase in cytokine load in patients with mTBI in comparison with healthy controls over a period of 3 months.^10^ Overall, few clinical studies have been conducted to evaluate the consequences of prolonged inflammation in TBI. Here, we show that patients with mTBI experience a state of systemic low-grade chronic inflammation, for up to 1 year after the initial injury, which is much longer than reported previously and independent of trauma beside the mTBI.

Almost all cytokines, which were increased in patients with mTBI in this study, have been reported to be associated with TBI. MCP-1 has been associated with post-concussion syndrome in mTBI patients.^10^ IL-8 (CXCL8) has been reported as a key mediator of neuroinflammation in severe TBI,^21^ eotaxin (CCL11) has been associated with chronic traumatic encephalopathy,^29^ and MIP-1β has been shown to be induced early in sports concussion.^30,31^ These chemokines induce production of the pro-inflammatory cytokine IFN-γ, which was increased in this study. IFN-γ has been shown to increase cell recruitment to an injured brain and thus local inflammation.^32,33^ Pro-inflammatory interleukins IL-17A^34^ and IL-9,^35^ as well as TNF^36^ are suggested to be involved in neuroinflammation after experimental severe (IL-17A and IL-9) and clinical repeated mTBI (TNF), by stimulating and controlling recruitment of neutrophils.^37^ To our knowledge, IL-9 has not been reported in clinical studies assessing TBI patients, yet. This may be due to pre-selection of known cytokines in previous studies. Given the large IL-9 elevations in patients with mTBI compared with controls in this study, further research into IL-9's relation to TBI is warranted. Growth factor FGF-basic has been shown to increase neurogenesis, preserve blood–brain barrier integrity, and enhance blood vessel proliferation, while suppressing autophagy.^38^

Notably, although each cytokine separately may be of limited pathophysiological importance for the clinical disease severity and outcome, and may differ substantially between individuals, the overall finding of sustained inflammation with an orchestra of many cytokines acting together and via different pathways might be of utmost importance. Crosstalk between the cytokines is frequently not detected when reductionistic systems such as isolated cells are used. We postulate that a holistic approach to understanding the neurobiological changes occurring in post-mTBI can be obtained only by using complex panels of assays in an *in vivo* situation, and that the possible implication to long-term neuroinflammation is a critical finding of our study that warrants further investigation.

### Associations between increased cytokine plasma levels and injury-related and demographic variables

There were several findings from the best-subset regression analyses that point to more severe injury being associated with lower levels of cytokines in blood at admission. First, eotaxin, MIP-1β, IL-9, and IP-10 were all negatively associated with traumatic intracranial MRI findings, indicating that in all cases, visible traumatic lesions resulted in lower systemic cytokine levels. This finding was unsuspected. It may suggest that systemic low-grade inflammation in patients with mTBI reflects disease severity not obviously linked to neuroradiological findings, a fact reported in other diseases such as febrile status epilepticus and brain swelling in cerebral malaria.^39,40^ Likewise, more severe injury indicated by lower GCS score was negatively associated with eotaxin and MIP-1β. Although GCS score has been shown to be an important predictor of morbidity in severe TBI,^41^ it has shown relatively poor prognostic utility in mTBI,^42^ and to our knowledge, its relation with inflammatory markers has not been investigated. Thus the significance of these findings remains to be explained.

On the other hand, LOC was positively associated with admission IFN-γ. IFN-γ is upregulated in the brain following TBI, but there is ongoing discussion about whether IFN-γ elevation is beneficial or detrimental.^43,44^ A large positive association of other injuries with TNF was demonstrated. These results are unsurprising, as TNF release has been reported in response to bone fractures and virtually all forms of ischemia/reperfusion injury in the acute phase.^45,46^ However, as TNF also was significantly increased in patients without other injuries at 3 and 12 months, TNF probably is associated with mTBI itself.

Age was associated with higher levels of the majority of cytokines: IL-8, eotaxin, MIP-1β, MCP-1, IL-17A, TNF, IL-1ra, IP-10, and PDGF. The impact of age on the immune system has been well characterized, and our results confirm that older individuals experience generally higher levels of inflammation.^47^

Lastly, sex showed different cytokine expression pattern in male compared with female patients. Although this phenomenon has been observed in several clinical and experimental studies in other diseases and is thought to be dependent on hormonal balances, our findings should be regarded as hypothesis generating.^48,49^

### Limitations

Inclusion of non-hospitalized patients led to considerable variation in time (up to 72 h) of obtaining the first “admission” blood sample, as many patients first needed to be tracked down and invited to participate. Thus, initial, time-critical cytokine kinetics immediately following the injury might have been lost. However, as systemic cytokine levels remained elevated and even increased during the observation period in some cases (IL-17A and FGF-basic), the initial kinetics and the significance thereof might be of less relevance in comparison with the long follow-up period.

As can be seen from Figures 1 and 2, we did not obtain blood samples from individuals at all time-points. Loss to follow-up is an issue that poses problems for generalizing group differences between early and late time-points. However, using a linear mixed model circumvents the majority of these issues with missing data.

Previous studies in TBI patients have reported absolute plasma cytokine levels, which differ to levels reported in this study.^31,50^ Methodologically, it is impossible to compare absolute cytokine levels when detection assays from different producers without standardized reference controls are used.^16^ Hence, absolute cytokine levels in this study should only be compared between patients with mTBI and the matched healthy controls, whereas pattern of expression and relative group differences are comparable between studies.^10^ In this study, only 11% of patients had MRI findings. Thus, regression analysis findings are based on relatively few patients and need to be verified in future studies.

### Implications for diagnosis, follow-up and therapy

The distinct pattern of cytokine expression observed in this study indicates a previously undocumented pattern of persistent inflammation following mTBI for 1 year post-injury. Factors normally used to grade severity of a TBI, such as GCS score, LOC, MRI findings, and PTA, were not associated consistently with cytokine expression patterns in this study. A biomarker especially for triage of patients with mTBI to guide diagnostic procedures is warranted; however, previous studies have demonstrated low specificity for inflammatory markers for predicting, for example, CT findings.^51^

Thus, further studies are needed to investigate if the demonstrated inflammatory state is caused by neuroinflammation, and what are the clinical implications of prolonged inflammation with outcome, such as post-concussion syndrome, and potential for manipulating it for improved outcome.

## Conclusion

This prospective observational study including clinic outpatients indicates that low-level systemic inflammation persists during 1 year post mild TBI as a high number of cytokines with different functions and etiologies remain increased through the observation period.

Long-term cytokine increase was not explained by injury severity determined by MRI findings or other injuries beside mTBI, which may imply that inflammation post mTBI may not solely be caused by trauma per se or obvious pathology in the brain tissue.

## Supplementary Material

Supplemental data

Supplemental data

Supplemental data

Supplemental data

Supplemental data
