# Supplementary material for: Systemic Inflammation Persists the First Year after Mild Traumatic Brain Injury: Results from the Prospective Trondheim Mild Traumatic Brain Injury Study
Source: J Neurotrauma. 2020 Sep 18;37(19):2120–30. doi: 10.1089/neu.2019.6963 (PMC7502683; doi:10.1089/neu.2019.6963)
Supplement: Supplemental data [file Supp_TableS1-S2.pdf]

Supplementary Table S1. Inclusion and Exclusion Criteria

Inclusion criteria (one or more)	*Exclusion criteria (one or more)*
Witnessed loss of consciousness (<30 min)	Non-residency in Norway
Confusion or disorientation	Non-fluency in Norwegian
Post-traumatic amnesia (<24 h)	Contraindications for MRI
Intracranial traumatic findings on head CT	Ongoing severe psychiatric,[a] neurological, or medical illness
Glasgow Coma Scale of 13–15 after 30 min post-injury or later upon presentation for healthcare	Presentation at clinic more than 48 h after trauma
	Concurrent major trauma (severe fractures, spinal cord or internal injuries)

[a]Severe psychiatric illness includes substance abuse.
CT, computed tomography; MRI, magnetic resonance imaging.

Supplementary Table S2. Results of a Log-Likelihood Test Comparing Mixed Models with and without Heterogeneous Variances for Each of the 12 Cytokines to Assess Whether Model Fit Was Significantly Improved

Cytokines	*Log likelihood ratio*	p-*value*
IFN-γ	103.23	**<0.0001**
IL-8	40.63	**<0.0001**
Eotaxin[a]	28.80	**<0.0001**
MIP-1β	54.90	**<0.0001**
MCP-1[a]	110.74	**≤0.0001**
IP-10[a]	14.68	**0.012**
IL-17A	65.94	**<0.0001**
IL-9	42.81	**<0.0001**
TNF	49.69	**<0.0001**
FGF-basic	69.87	**<0.0001**
PDGF	31.16	**<0.0001**
IL-1ra[a]	26.34	**≤0.0001**

[a]Log transformed data.
FGF-basic, basic fibroblast growth factor; IL, interleukin; IL-1ra, IL-1 receptor antagonist; IFN-γ, interferon gamma; IP-10, IFN-γ-inducing protein 10; MCP-1, monocyte chemoattractant protein 1; MIP-1β, macrophage inflammatory protein-1-beta; PDGF, platelet-derived growth factor; TNF, tumor necrosis factor.
