# Supplementary material for: Systemic Inflammation Persists the First Year after Mild Traumatic Brain Injury: Results from the Prospective Trondheim Mild Traumatic Brain Injury Study
Source: J Neurotrauma. 2020 Sep 18;37(19):2120–30. doi: 10.1089/neu.2019.6963 (PMC7502683; doi:10.1089/neu.2019.6963)
Supplement: Supplemental data [file Supp_TableS3.pdf]

Supplementary Table S3. Results of All-Subsets Multiple Linear Regression Using Each of the 12 Cytokines as Outcome Variables and All Demographic and Clinical Variables Listed in the Left of the Table as Possible Predictors

Acute cytokine concentrations:β-value, [95% CI], p-*value*

	IFN-γ	*IL-8*	*Eotaxin*[a]	*MIP-1β*	*IL-17A*	*IL-9*	*TNF*	*FGF-basic*	*IL-1ra*[a]	*MCP-1*†	*IP-10*†	*PDGF*
Sex: Female[b]	0.8 [-0.3 – 1.9] $p=0.150$		-0.1 [-0.2 – 0.02] $p=0.129$		2.9 [-5.9 – 0.1] $p=0.055$			-6.4 [-12.7 – -0.1] **$p = 0.048$**				-74.7 [-161.0 – 11.6] $p=0.089$
Age		0.1 [-0.01 – 0.2] $p=0.076$	.003 [0.001 – 0.007] **$p = 0.03$**	0.3 [-0.1 – 0.6] $p=0.094$	0.1 [-0.02 – 0.2] $p=0.101$		0.2 [-0.03 – 0.4] $p=0.099$		0.006 [-001 – 0.013] $p=0.070$	0.003 [-0.001 – 0.009] $p=.122$	0.004 [0.001 – 0.007] **$p = 0.007$**	2.9 [-0.3 – 6.1] $p=0.073$
GCS: 13-14[c]			-0.1 [-0.2 – 0.006] $p=0.063$	-11.9 [-23.5 – -0.2] **$p = 0.046$**								
LOC: Known[d]	1.2 [0.2 – 2.3] **$p = 0.025$**			-7.2 [-16.3 – 1.8] $p=0.118$								-62.7 [-147.1 – 21.7] $p=0.144$
PTA: 1-24 h[e]												
MRI finding: Yes[f]			-0.2 [-0.4 – -0.1] **$p = 0.003$**	-18.7 [-33.1 – -4.3] **$p = 0.011$**		-13.6 [-26.7 – -0.5] **$p = 0.043$**					-0.3 [-0.4 – -0.1] **$p = 0.0001$**	
Other injuries: Yes[g]			-0.1 [-0.2 – 0.02] $p=0.133$				8.5 [2.7 – 14.2] **$p = 0.004$**					

[a]Log transformed data. [b]Baseline comparison for Sex was male. [c]Baseline comparison for GCS scores of 13-14 was GCS score of 15.

[d]Baseline comparison for Known LOC was ''Unknown LOC.'' [e]Baseline comparison for PTA duration of between 1 and 24 h was PTA of less than 1 h. [f]Baseline comparison for MRI findings was ''No MRI finding.'' [g]Baseline comparison for Other injuries was ''No Other Injuries.'' Empty spaces indicate the best model fit did not include that association. Beta values are non-standardized. Significant p-values <0.05 are bolded.

FGF-basic, basic fibroblast growth factor; GCS, Glasgow Coma Score; IL, interleukin; IL-1ra, IL-1 receptor antagonist; IFN-γ, interferon gamma; IP-10, IFN-γ-inducing protein 10; LOC, loss of consciousness; MCP-1, monocyte chemoattractant protein 1; MIP-1β, macrophage inflammatory protein-1-beta; MRI, magnetic resonance imaging; PDGF, platelet-derived growth factor; PTA, post-traumatic amnesia; TNF, tumor necrosis factor.
