# Supplementary material for: Systemic Inflammation Persists the First Year after Mild Traumatic Brain Injury: Results from the Prospective Trondheim Mild Traumatic Brain Injury Study
Source: J Neurotrauma. 2020 Sep 18;37(19):2120–30. doi: 10.1089/neu.2019.6963 (PMC7502683; doi:10.1089/neu.2019.6963)
Supplement: Supplemental data [file Supp_TableS4.pdf]

Supplementary Table S4. Means and Standard Deviations of all Cytokines, Grouped by Dichotomous Injury-Related and Demographic Characteristics.

	IFN-γ *Mean ± SD*	*IL-8* *Mean ± SD*	*Eotaxin*[a] *Mean ± SD*	*MIP-1β* *Mean ± SD*	*IL-17A* *Mean ± SD*	*IL-9* *Mean ± SD*	*TNF* *Mean ± SD*	*FGF-basic* *Mean ± SD*	*IL-1ra*[a] *Mean ± SD*	*MCP-1*[a] *Mean ± SD*	*IP-10*[a] *Mean ± SD*	*PDGF* *Mean ± SD*
Sex:												
Male	3.4 ± 3.4	9.5 ± 10.5	48.7 ± 52.9	99.7 ± 33.3	12.9 ± 10.9	51.9 ± 29.9	37.5 ± 21.2	30.9 ± 23.0	189.2 ± 201.3	19.2 ± 20.9	372.2 ± 348.8	382.1 ± 332.3
Female	4.3 ± 4.3	8.3 ± 9.4	42.5 ± 50.2	96.1 ± 29.8	10.3 ± 8.5	48.3 ± 26.3	35.1 ± 16.2	25.2 ± 17.7	183.8 ± 163.3	18.3 ± 24.2	352.7 ± 315.9	292.7 ± 230.3
GCS:												
15	3.6 ± 3.8	9.4 ± 10.5	49.8 ± 55.8	100.5 ± 32.4	11.9 ± 10.5	51.3 ± 29.5	36.7 ± 20.0	28.3 ± 21.5	187.0 ± 197.1	20.1 ± 23.1	371.9 ± 353.2	337.1 ± 288.1
13-14	4.7 ± 3.6	9.3 ± 8.7	35.5 ± 38.4	85.8 ± 30.1	12.5 ± 9.4	46.1 ± 26.3	35.2 ± 18.6	31.1 ± 21.8	210.1 ± 165.8	16.5 ± 20.5	318.2 ± 250.5	373.6 ± 330.8
LOC:												
Unknown	3.3 ± 3.4	9.8 ± 11.2	45.7 ± 49.0	102.2 ± 34.1	12.4 ± 11.4	52.4 ± 29.4	37.7 ± 21.4	28.7 ± 22.5	193.6 ± 223.0	18.5 ± 20.9	365.4 ± 344.1	374.9 ± 319.9
Known	4.3 ± 4.1	8.2 ± 8.6	47.0 ± 55.3	93.8 ± 28.9	11.4 ± 8.4	48.5 ± 27.6	35.3 ± 16.9	28.9 ± 20.0	179.6 ± 135.7	19.2 ± 23.7	364.1 ± 328.4	317.6 ± 274.8
PTA:												
< 1 h	3.7 ± 3.8	9.3 ± 10.9	50.3 ± 56.3	101.5 ± 33.6	12.0 ± 10.6	53.0 ± 30.4	37.2 ± 21.1	28.5 ± 21.9	176.3 ± 139.9	20.6 ± 23.1	384.0 ± 360.9	359 ± 304.6
1-24 h	4.0 ± 3.5	8.3 ± 7.9	37.0 ± 38.5	90.9 ± 26.9	11.8 ± 8.9	44.9 ± 23.2	35.4 ± 15.1	29.3 ± 20.1	212.2 ± 267.0	14.7 ± 19.4	320.4 ± 267.5	324 ± 291.8
MRI finding:												
No	3.8 ± 3.5	9.3 ± 10.5	49.2 ± 54.1	100.6 ± 32.1	12.1 ± 10.4	52.0 ± 29.3	37.2 ± 20.3	28.8 ± 21.9	187.5 ± 195.6	19.8 ± 23.3	386.0 ± 347.9	353.8 ± 306.1
Yes	3.8 ± 5.6	6.5 ± 4.7	21.9 ± 7.8	78.5 ± 23.3	10.8 ± 7.8	38.3 ± 18.3	31.8 ± 9.5	28.1 ± 15.4	184.3 ± 97.1	11.0 ± 3.7	185.7 ± 96.9	302.6 ± 250.0
Other injuries:												
No	3.8 ± 3.8	9.2 ± 10.1	51.1 ± 58.6	98.2 ± 32.8	11.9 ± 9.1	49.7 ± 29.8	33.4 ± 14.2	27.9 ± 20.6	181.8 ± 203.6	20.2 ± 23.8	372.6 ± 363.9	336.8 ± 284.0
Yes	3.7 ± 3.6	8.7 ± 10.0	38.3 ± 36.9	98.6 ± 30.8	12.0 ± 11.6	52.0 ± 26.6	42.1 ± 25.3	30.3 ± 22.5	196.2 ± 157.4	16.5 ± 19.0	351.7 ± 284.8	368.0 ± 327.7

[a]Data are not log-transformed.

Age was not included as it is not a dichotomous variable. Colored backgrounds correspond to those in Figure 5, that is, all colored backgrounds are interactions included in the best model, as assessed using an all-subsets regression, with each cytokine input as outcome variables, and all other variables (left-hand column) included as possible predictors. A white background indicates that the predictor was not included in the final model. The direction and size of regression coefficient is represented according to the figure legend color scheme of Figure 5, whereby increasingly positive associations are graded to red, and increasingly negative associations are graded to blue.

FGF-basic, basic fibroblast growth factor; GCS, Glasgow Coma Score; IL, interleukin; IL-1ra, IL-1 receptor antagonist; IFN-γ, interferon gamma; IP-10, IFN-γ-inducing protein 10; LOC, loss of consciousness; MCP-1, monocyte chemoattractant protein 1; MIP-1β, macrophage inflammatory protein-1-beta; MRI, magnetic resonance imaging; PDGF, platelet-derived growth factor; PTA, post-traumatic amnesia; SD, standard deviation; TNF, tumor necrosis factor.
