# Supplementary material for: Systemic Inflammation Persists the First Year after Mild Traumatic Brain Injury: Results from the Prospective Trondheim Mild Traumatic Brain Injury Study
Source: J Neurotrauma. 2020 Sep 18;37(19):2120–30. doi: 10.1089/neu.2019.6963 (PMC7502683; doi:10.1089/neu.2019.6963)
Supplement: Supplemental data [file Supp_TableS5-S6.pdf]

Supplementary Table S5. Summary of Main Effects of Group (patients with mTBI without other injuries, compared with controls), Time, and the Interaction of Group and Time, for the Cytokines TNF and Eotaxin, Which Showed Significant Effects of Other Injuries in the Best-Subset Multiple Regression Analyses

Cytokines	*Group F-value* (p-*value*)	*Time F-value* (p-*value*)	*Interaction (Group-by-Time) F-value* (p-*value*)
TNF	F = 11.09, ***p* = 0.001**	F = 8.44, ***p* = 0.0003**	F = 2.36, *p* = 0.096
Eotaxin[a]	F = 8.67, ***p* = 0.004**	F = 1.27, *p* = 0.283	F = 0.67, *p* = 0.510

[a]Log transformed data. Significant differences are bolded.

Group: patients with mTBI without other injuries, compared with controls; Time: time course of cytokine concentrations when group is not taken into account; Group-by-Time: interaction of Group and Time; a significant effect indicates the time courses of patients with mTBI and community controls significantly differed.

mTBI, mild traumatic brain injury; TNF, tumor necrosis alpha.

Supplementary Table S6. Group Comparisons between Patients with mTBI without Other Injuries and Controls at Each Time-Point, for TNF and Eotaxin, Which Showed Significant Effects of Other Injuries in the Best-Subset Multiple Regression Analyses

	Admission Estimate[a] *[95% CI]* p-*value*	*3 months Estimate*[a] *[95% CI]* p-*value*	*12 months Estimate*[a] *[95% CI]* p-*value*
TNF	4.64 [-0.09 – 9.37] *p* = 0.055	8.60 [3.85 – 13.35] ***P* = 0.0004**	8.57 [3.56 – 13.58] ***P* = 0.0009**
Eotaxin[b]	**0.12** [0.03 – 0.21] ***p* = 0.011**	**0.14** [0.05 – 0.23] ***p* = 0.003**	**0.15** [0.05 – 0.24] ***p* = 0.003**

[a] Estimate refers to mean group differences as estimated by the mixed model. CI is the 95% confidence interval of the estimated group difference.

[b]Log transformed data. Significant differences are bolded.

mTBI, mild traumatic brain injury; TNF, tumor necrosis factor.
